# Supplementary material for: Core proteome mediated subtractive approach for the identification of potential therapeutic drug target against the honeybee pathogen Paenibacillus larvae
Source: PeerJ. 2024 May 27;12:e17292. doi: 10.7717/peerj.17292 (PMC11138523; doi:10.7717/peerj.17292)
Supplement: Supplemental Information 1 [file peerj-12-17292-s001.docx]

| **Supplementary Table 1.** List of 24 essential *P. larvae* proteins determined by DEG and BLASTp software. | | | | | | |
| --- | --- | --- | --- | --- | --- | --- |
| **Protein name** | **DEG identifier** | **Coverage of the request** | **E-value** | **Identity percentage** | **Length** | **Accession** |
| 1-ATP-dependent helicase | DEG10550152 | 9% | 9e-07 | 35.59% | 578 | WP_104932911.1 |
| 2-helicase-exonuclease AddABsubunitAddA | DEG10050476 | 14% | 1e-09 | 37.23% | 1391 | WP_042119081.1 |
| 3-amino acidadenylationdomain-containingprotein | DEG10470206 | 55% | 0.0 | 74.10% | 776 | WP_237088672.1 |
| 4-sigma-70 family RNA polymerase sigma factor | DEG10360071 | 43% | 1e-06 | 30.56% | 114 | WP_052337356.1 |
| 5-asparagine--tRNA ligase | DEG10370076 | 98% | 1e-172 | 52.61% | 432 | WP_024093784.1 |
| 6-UvrD-helicase domain-containing protein | DEG10560996 | 6% | 7e-11 | 42.68% | 120 | WP_024095352.1 |
| 7-acyltransferasedomain-containingprotein | DEG10270361 | 8% | 8e-28 | 38.66% | 193 | WP_237088801.1 |
| 8-PD-(D/E)XK nuclease family protein | DEG10580098 | 2% | 0.042 | 59.09% | 516 | WP_237089125.1 |
| 9-chromosome segregationprotein SMC | DEG10070066 | 66% | 9e-116 | 36.09% | 1192 | WP_023484350.1 |
| 10-2,4-dienoyl-CoA reductase | DEG10470353 | 98% | 2e-135 | 75.30% | 254 | WP_079940690.1 |
| 11-iron-containing alcoholdehydrogenase, partial | DEG10430416 | 37% | 1e-36 | 52.03% | 166 | WP_192807335.1 |
| 12-UvrD-helicase domain-containing protein, partial | DEG10580104 | 4% | 4e-04 | 40.98% | 316 | WP_040931833.1 |
| 13-ketoacyl-ACP synthase III | DEG10570076 | 97% | 7e-83 | 49.39% | 325 | WP_174567582.1 |
| 14-condensation domain-containingprotein, partial | DEG10470515 | 53% | 3e-162 | 39.62% | 803 | WP_040931378.1 |
| 15-acetyl-CoA C-acyltransferase | DEG10250448 | 9% | 7e-04 | 50.00% | 394 | WP_042118900.1 |
| 16-ACP S-malonyltransferase | DEG10010117 | 99% | 7e-119 | 54.60% | 311 | WP_023484345.1 |
| 17-(2,3-dihydroxybenzoyl) adenylate synthase | DEG10270431 | 96% | 1e-138 | 44.01% | 541 | WP_083038287.1 |
| 18-isochorismatase | DEG10470205 | 100% | 2e-158 | 69.90% | 308 | WP_024093894.1 |
| 19-DNA helicasePcrA | DEG10010080 | 100% | 0.0 | 58.66% | 776 | WP_077996486.1 |
| 20-SDR familyoxidoreductase | DEG10470172 | 68% | 2e-28 | 38.69% | 250 | WP_024094242.1 |
| 21-non-ribosomal peptide synthetase | DEG10470206 | 99% | 0.0 | 67.23% | 2387 | WP_172423458.1 |
| 22-beta-ketoacyl-ACP synthase II | DEG10560281 | 99% | 1e-146 | 52.31% | 411 | WP_024094309.1 |
| 23-beta-ketoacyl-ACP synthase II, partial | DEG10010090 | 88% | 1e-172 | 62.84% | 365 | WP_040930630.1 |
| 24-N-acetylmuramoyl-L-alanine amidase | DEG10470083 | 16% | 7e-13 | 38.54% | 97 | WP_198963601.1 |


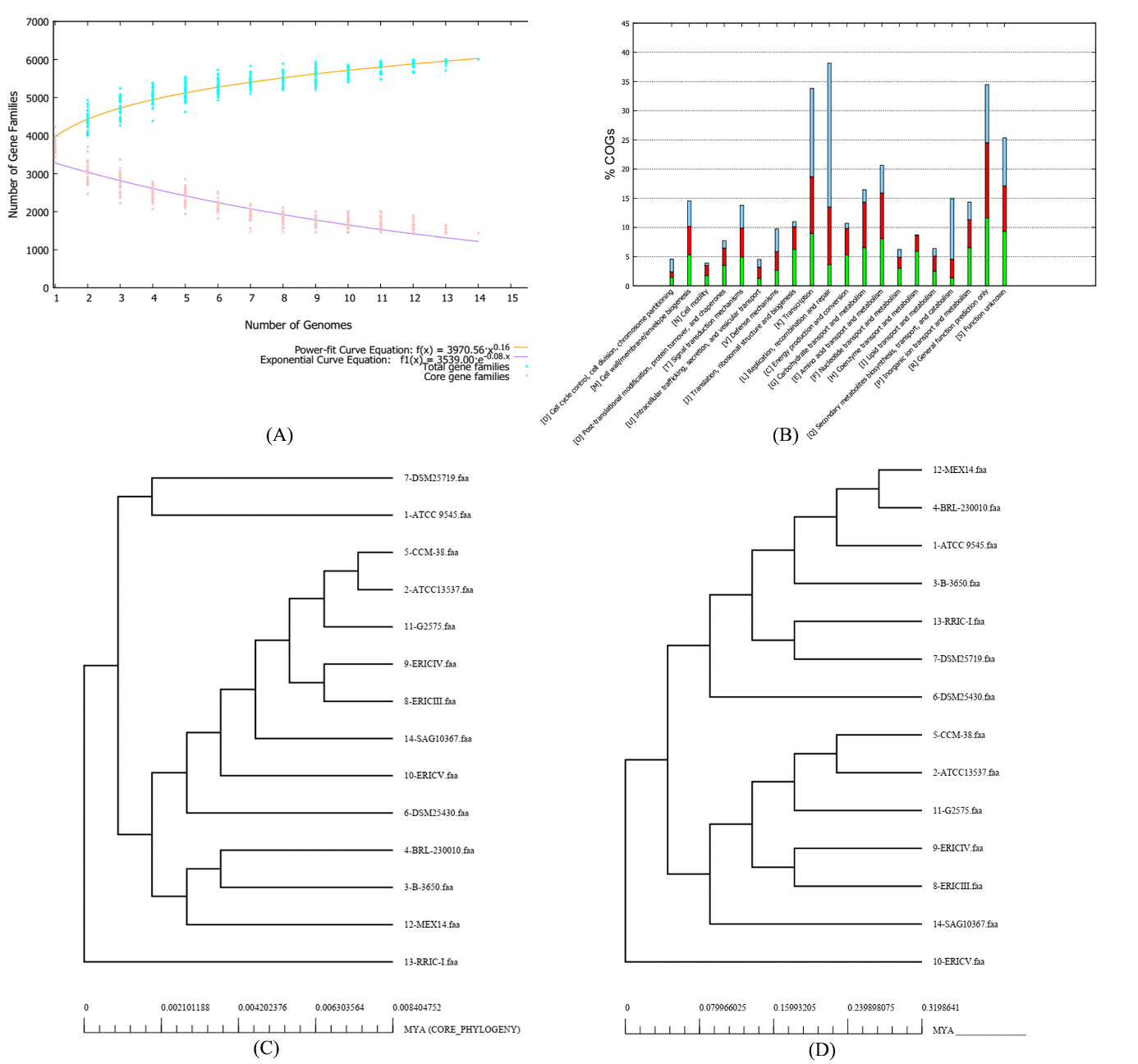


Supplementary Fig. 1. (A) Core-pan proteome dot plot of studied strains. (B) Percent COGs shown for studied strains in respective genome fractions. Core fraction shown by green, accessory by red and unique by sky blue. (C)Core proteome based phylogenetic tree of studied strains (D) Pan-proteome based phylogeny of studied strains.


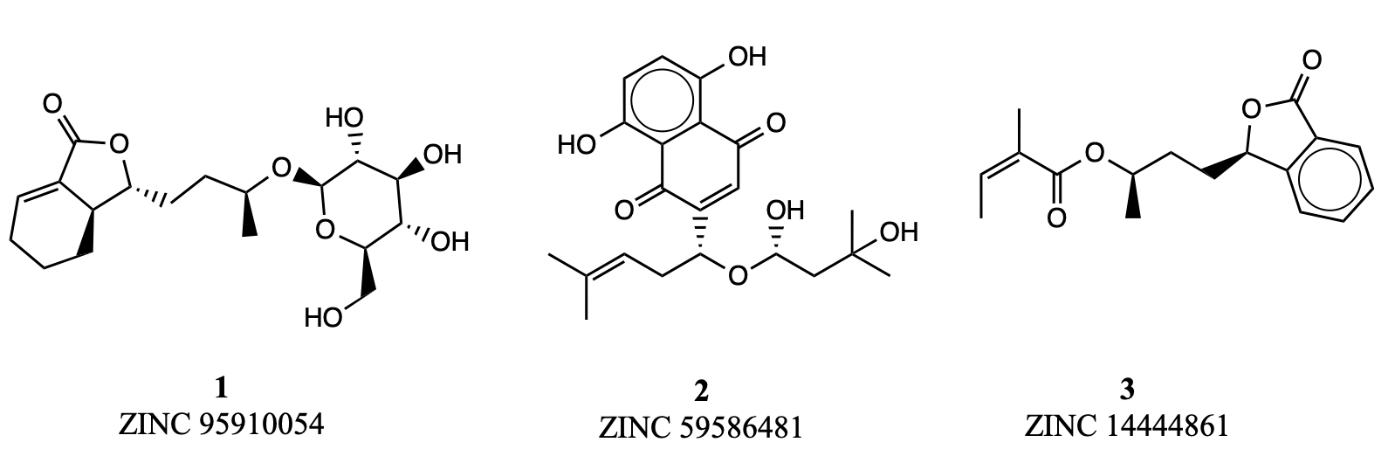


Supplementary Fig. 2. 2D structures of top scoring ligands.
