# Supplementary material for: Core proteome mediated subtractive approach for the identification of potential therapeutic drug target against the honeybee pathogen Paenibacillus larvae
Source: PeerJ. 2024 May 27;12:e17292. doi: 10.7717/peerj.17292 (PMC11138523; doi:10.7717/peerj.17292)
Supplement: Supplemental Information 2 [file peerj-12-17292-s002.docx]

**Supplemental File: Explanation for Addition of New Authors**

*Manuscript Title: [Core proteome mediated subtractive approach for the identification of potential therapeutic drug target against the honeybee pathogen Paenibacillus larvae]*

*Submission ID: [(#2023:10:92437:1:0:CHECK)]*

*Date: [16-01-2024]*

Dear PeerJ Editorial Team,

I am providing this supplemental file to offer detailed and valid reasons for the addition of new authors to our manuscript titled "[ *Core proteome mediated subtractive approach for the identification of potential therapeutic drug target against the honeybee pathogen Paenibacillus larvae*]." The inclusion of Hanen Najjaa, an expert in the valorization of Medicinal and Aromatic Plants, and Salim Lebbal, an entomologist, and Calvin R expert in MD simulation study, is based on their substantial contributions aligned with their specific areas of expertise.

**Category 1: Substantial Contributions to Conception, Design, and Execution**

Both authors bring unique expertise, with Hanen Najjaa specializing in the valorization of Medicinal and Aromatic Plants and Salim Lebbal contributing as an expert in bee life and entomology.

**Conception and Design:**

- - *Hanen Najjaa (hanen.najjaa@yahoo.fr):* Spearheaded the virtual screening process, leveraging ligands extracted from the Chinese medicine database (n=36,043), ensuring the selection of diverse and relevant phytomolecules aligned with the valorization of Medicinal and Aromatic Plants.
  - *Salim Lebbal (salim-leb@hotmail.com):* Contributed to the conception and design phase, providing valuable insights as an entomologist with expertise in bee life. Salim Lebbal's input influenced the choice of subject matter, ensuring its relevance and significance.

**Execution - Virtual Screening:**

- - *Hanen Najjaa (hanen.najjaa@yahoo.fr):* Led the execution of virtual screening, utilizing the AutoDockVina software, known for its accurate prediction of binding affinity. She oversaw the addition of missing hydrogens, conversion of protein and ligand to pdbqt format, and the meticulous definition of docking box parameters.

**Execution – MD simulation:**

- - Calvin R Wei led the execution of MD simulation conducted using Gromacs.

**Analysis and Visualization:**

- - *Hanen Najjaa:* Contributed significantly to the analysis of virtual screening results, ranking screened ligands by binding energy and identifying potential enzyme inhibitors. Visualization of interactions was done using Maestro v2020-3 (Schrodinger LLC).
  - *Calvin R Wei :* contribute to the analysis of the MD simulation result.

**Drafting and Critical Revision:**

- - *Salim Lebbal:* Actively involved in drafting and revising the manuscript, providing critical intellectual content, especially in discussion part.
  - *Hanen Najjaa:* Played a pivotal role in the critical revision of the manuscript
  - *Calvin R Wei:* Played an integral role in meticulously reviewing and revising the manuscript.

We acknowledge that it was a mistake not to include Hanen Najjaa and Salim Lebbal and Calvin R Wei from the beginning. Their expertise significantly contributes to the success and relevance of our manuscript. We appreciate your understanding of this oversight.

This supplemental file aims to highlight the diverse expertise of both authors, and we believe their unique perspectives enhance the overall quality and scope of our work.

We appreciate your thorough review and understanding of these additions. Should you require further clarification or information, please do not hesitate to contact us promptly.

Thank you for your time and consideration.

Sincerely,
